# Twins; Footling Presentation

**Published:** 1844-08-10

**Authors:** 


### TWINS; FOOTLING PRESENTATION;

*Wedging of the two heads, neck and head together, in the pelvis; artificial delivery, with loss of first child; recovery of mother, and safety of second child.*

At seven in the morning the membranes had given way, and the feet of a fœtus almost immediately followed the gush of liquor amnii. The midwife in attendance proceeded at once to disengage the child; but before she could get it away a very violent pain occurred, with unusual suffering referred to the cavity of the pelvis. The infant, born all save the head, could not be brought farther, and the pains only recurring at intervals of a quarter of an hour soon died away entirely. Assistance was sought at twelve, and again at three o'clock in the day. The body of the half-born fœtus was cold, darkly livid, without pulsation, and had evidently been lifeless for many hours. Every attempt to get it away proved vain; it was in fact now ascertained that with the head belonging to the fœtus that was partially born, there was the head of a second fœtus interlocking, which rendered every prospect of effecting the delivery nugatory. The strength of the mother began to fail, and there was nothing for it but to proceed to the apparently barbarous but indispensable process of severing the body from the head of the dead fœtus. This was done, and the head was then readily pushed back into the cavity of the uterus. No pains supervening, the forceps were applied, and the second infant was brought into the world alive. But now profuse hæmorrhage set in, which was only controlled by injections of pure brandy. The grand object was now to extract the head, in which great difficulty was experienced; at length, however, it was caught between the blades of the forceps, and being brought down within reach of the finger, this was got into the mouth, and the delivery accomplished. The placenta soon followed. The mother and second child did well.—*Dr. Hoffmann, in Casper's Wochenschrift.*—*London Med. Gaz.*

